# A novel approach for joint indoor localization and activity recognition using a hybrid CNN-GRU and MRF framework

**DOI:** 10.1371/journal.pone.0328181

**Published:** 2025-08-07

**Authors:** Sarmad Sohaib, Syed Mohsin Bokhari, Muhammad Shafi, Anas Alhashmi

**Affiliations:** 1 Department of Electrical and Electronic Engineering, University of Jeddah, Jeddah, Saudi Arabia; 2 Department of Electrical and Computer Engineering, University of Engineering and Technology, Taxila, Pakistan; 3 School of Computing, Ulster University, Belfast, United Kingdom; Chunghwa Telecom Co. Ltd., TAIWAN

## Abstract

This work proposes a new hybrid model for joint indoor localization and activity recognition by combining a Convolutional Neural Network-Gated Recurrent Unit (CNN-GRU) model with a Markov Random Field (MRF) for better classification. The CNN-GRU successfully captures spatial and temporal dependencies, while the MRF models the mutual relations of activities and locations by estimating their joint probability distribution. The new system was tested on a public smart home dataset with four activities (sitting, lying, walking, and standing) and four indoor locations (kitchen, bedroom, living room, and stairs). The hybrid framework obtained an accuracy of 95% for activity recognition and 93% for indoor localization with a combined activity-location classification accuracy of 81%. Such results confirm the ability of the system to provide robust predictions in real-world smart environments, make it highly suitable for healthcare and intelligent living applications, and is efficient and deployable in real-world scenarios, addressing the critical challenges of noisy and dynamic indoor environments.

## 1 Introduction

The joint localization and recognition of human activity is a vital and developing research area, as information regarding human locations and actions is essential for numerous applications, including intelligent healthcare systems, smart homes, physical training, surveillance, human-computer interaction, and environmental awareness. The sophisticated healthcare system must monitor the real-time whereabouts of older people and oversee their activities to facilitate independent living while mitigating the danger of falls and accidents. Conventional techniques for recognizing human actions rely on analyzing inertial measurement unit sensor data and computer vision approaches [1]. Cheng *et al*. [2] introduced several approaches for detecting human body activities using wearable acceleration sensors, yielding favorable identification outcomes. Motion signals based on magnetic induction were used in [3] to identify human activities. However, accelerometers cannot deliver precise long-term location data owing to measurement inaccuracies and drift, and magnetometers are susceptible to interference from changing ambient magnetic fields. A vision-based approach was used for action recognition and localization in [4], with promising results. Nonetheless, vision-based systems include drawbacks related to privacy violations and susceptibility to lighting changes and obstructions, limiting their use in everyday scenarios. Indoor positioning systems have garnered more interest in recent years. Diverse technologies, such as radio frequency identification (RFID), optical light, WiFi, ultrasound, Bluetooth, and ultra-wideband (UWB), have been used in indoor positioning systems, with some technologies employed for human activity detection and localization. A WiFi fingerprinting method for activity detection and indoor localization was presented in [5]. This technique can identify only a limited number of locations inside interior environments since WiFi fingerprinting relies on Received Signal Strength (RSS) and often attains meter-level precision. Among the previously described technologies, UWB is one of the most promising indoor positioning systems because it can achieve centimeter-level precision, resilience to multi-path fading and interference, and low power consumption [6,7].

Channel state information (CSI) of WiFi devices has been widely studied for human sensing applications, such as activity detection [8], gesture recognition, indoor localization [9], and healthcare [10]. The success is due to many unique features of WiFi, such as the pervasive deployment of commercial WiFi devices, immunity to lighting conditions and obstacles beyond the limit of cameras, and non-intrusive sensing without requiring extra effort from users. While there is much research on the specific task of WiFi human sensing, there is little focus on the integrated activity detection and indoor localization problem. Accomplishing the integrated task will give rise to several favorable human-computer interaction applications. In a smart home with Internet-of-Things (IoT) devices, the gadgets may show different reactions to the same gesture commands depending on the location of the users [11].

More recent research has sought hybrid sensor fusion techniques to mitigate indoor localization noise. For instance, Brena *et al*. [12] proposed an adaptive fusion technique based on different technologies such as Wi-Fi RSSI, inertial sensors, and magnetic field signals, which are time-synchronized among themselves through a Kalman filter for improving localization precision. Their technique dynamically adapts to environmental changes and user mobility, effectively mitigating uncertainties caused by signal fluctuations and obstructions. Such approaches highlight the importance of probabilistic filtering and context-aware adaptation to facilitate robust localization, which is especially beneficial for systems such as ours that require accurate user positioning as a precursor to activity recognition.

Similarly, Candeloro *et al*. [13] developed a hybrid deep learning architecture that combines CNNs for spatial feature extraction and LSTMs for temporal modeling to tackle challenges caused by noisy sensor readings and unpredictable human motion in indoor localization. Their approach performed robustly under changing conditions caused by movement and signal dynamic environments.However, unlike Candeloro *et al*., [13] our contribution includes a Markov Random Field (MRF) to jointly model the relationship between activities and locations, extending beyond localization alone. Nevertheless, combining more advanced noise-robust models is a promising direction to expand our framework’s applicability to uncontrolled, real-world deployments.

This study deals with joint indoor localization and activity recognition by introducing a Hybrid Convolutional Neural Network Gated Recurrent Unit (CNN-GRU) model for the classification of predefined locations and activities and then applying a Markov Random Field (MRF) to model the mutual relations between locations and activities by estimating their joint probability. We focus on the classification of four daily activities: sitting, lying, walking, and standing. In addition, we classify four indoor locations: kitchen, bedroom, living room, and stairs. To evaluate the effectiveness of our proposed models, we present the results using the necessary performance metrics, such as the confusion matrix, F1 score, precision, and recall. The proposed approach enhances joint prediction accuracy while ensuring seamless deployment in real-world scenarios.

The key contributions of this work are as follows:

- **Hybrid CNN-GRU model:** The proposed approach utilizes a hybrid CNN-GRU model to individually classify locations and activities while capturing spatial and temporal dependencies. Such an architecture allows effective modeling of dynamic activity patterns with location information.
- **Joint activity-location classification:** A novel aspect of the research uses a combined activity-location classification approach, where the outputs from separate activity and location models are fused to predict activity-location pairs. This integration leads to more accurate predictions compared to independent classification.
- **Markov random field (MRF) integration:** Introducing the Markov Random Field (MRF) framework allows for modeling dependencies among activities and locations. MRF effectively refines the joint prediction and improves the consistency among predicted activity-location pairs through the edge potential matrix encoding compatibility between activities and locations.
- **Improved inference and accuracy:** The integrated approach of CNN-GRU and MRF enhances the overall inference. The methodology, by maximizing the joint probability *P*(*a*,*l*) using the MRF, yields the most probable and consistent activity-location pairs for increased classification accuracy and decreased errors in prediction.
- **Enhanced real-world applicability:** The methodology exhibits real-world applicability in critical activity recognition and localization scenarios. The model can be applied to various domains, such as healthcare, smart homes, and location-based services, where activity and localization must be identified simultaneously.

The subsequent sections of this paper are organized as follows. Sect 2 outlines the related work in literature. Materials and methods employed in the proposed approach are presented in Sect 3. Sect 4 presents the proposed FLPMDP algorithm to diagnose arrhythmia. Sect 5 describes the experimental setup, presenting the results and their interpretation as the empirical basis. Finally, Sect 6 concludes the paper, summarizing the key findings and suggesting directions for future research.

## 2 Related work

In recent years, many efforts have been devoted to activity recognition and indoor localization using a wide range of methodologies and sensor modalities. In the work by Bock *et al*. [14], the authors presented temporal action localization models for inertial-based human activity recognition and showcased improved performance over classical inertial models. Likewise, Zandi *et al*. [15] introduced RoboFiSense, a novel framework for WiFi sensing-based robotic arm activity classification, which underlines the capability of non-invasive sensing. Pagan *et al*. [16] introduced an ultra-low-power activity recognition system with adaptive compressed sensing and highlighted energy-aware solutions for remote health monitoring. Neural network architecture advances have been instrumental in enhancing activity recognition, as seen in spatio-temporal graph convolutional networks introduced in [17], which nicely capture spatial and temporal dependencies from activity data. Moreover, self-supervised learning techniques [18] have decreased dependence on labeled data while boosting generalization in wearable sensor-based recognition tasks.

Of these, innovating multimodal approaches has been particularly striking. For example, Konak *et al*. [19] introduced real-time 2D pose estimation for optimal sensor placement in activity recognition, which fills the gap between video-based and sensor-based approaches. Similarly, [20] and HiFi-Net++ [21] underline the application of large language models and hierarchical fine-grained detection techniques to improve interpretability and robustness in localization tasks. Cross-domain challenges have also been addressed in recent works, with transfer learning approaches [22] allowing models to adapt across diverse datasets, thereby overcoming the constraints of domain-specific training. IoT-enabled frameworks, such as those presented in [23], allow for a robust analysis of sensor data for joint activity recognition and localization. WiFi sensing systems like in [15] underline the application of channel state information (CSI) for non-invasive activity classification and transition toward infrastructure-independent human activity recognition (HAR) solutions.

Deep learning has dramatically improved the performance of HAR by efficiently exploiting multimodal sensor data. Methods based on Convolutional Neural Networks (CNNs) and Spatio-Temporal Graph Convolutional Networks (ST-GCNs) [24] have achieved better recognition accuracy by modeling spatio-temporal dependencies. Transfer learning techniques [25] deal with cross-domain issues, allowing activity recognition across different datasets. Self-supervised learning frameworks [18] have also alleviated the reliance on labeled data and made HAR more scalable. Energy efficiency is still an essential aspect of IoT-based and wearable systems. Adaptive compressed sensing frameworks [22] would significantly decrease the cost of data transmission while achieving high recognition accuracy, which is especially valuable in applications of remote health monitoring, where power consumption is a very critical issue. Integration of attention mechanisms and noise-assisted models, as in AdaIFL [26], has improved localization and activity recognition in dynamic environments. Multi-modal approaches, e.g., wearable sensors and WiFi signals [27], give holistic solutions to the challenges in HAR and localization. Moreover, Graph Neural Networks (GNNs) [28] have been exploited to model complicated spatial temporal relationships in human activity recognition with superior accuracy and adaptability over conventional neural networks.

## 3 Problem formulation and dataset

**Fig 1:**
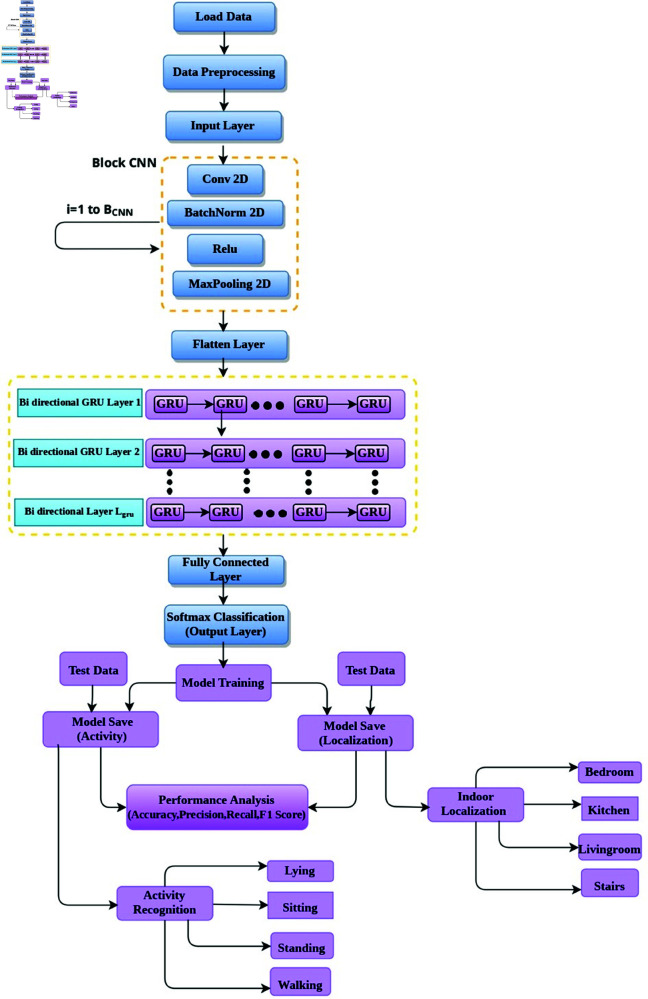
The proposed framework of hybrid CNN-GRU for joint classification of indoor localization and activity recognition.

This section presents the proposed research methodology, including a description of the chosen dataset, its preprocessing, and the architectures of the proposed models. The proposed framework for joint classification of localization and activity recognition is shown in Fig 1.

### 3.1 Problem formulation

The growing demand for context-aware applications in such systems as smart homes, health monitoring, and location-based services calls for accurate and reliable algorithms for indoor localization and activity recognition. While considerable progress has been made in these individual fields, the unification of activity detection and localization into a single framework is still a big challenge. This is due to the intricate interaction of spatial and temporal characteristics in activities and locations, along with their mutual relations.

More importantly, many of the recent approaches suffer from scalability, robustness, and practical usability issues, especially in dynamic and noisy indoor settings. These problems bring to light the requirement for a holistic approach that not only explains the interrelations between activity and place but also allows effective and successful joint classification.

### 3.2 Data preparation

This research use a publicly accessible dataset [29] collected in a smart home environment to evaluate our proposed method. Data was gathered in a two-bedroom, two-story terraced house in a residential area using the EurValve SHiB system [30]. The system comprises one wearable wrist device, four gateways placed in strategic locations (living room, kitchen, bedroom, and upstairs staircase landing), and a 4G network for sending data to a central server for processing. The wearable device contains a tri-axial ADXL362 accelerometer with a range of ±4g, sampling accelerometer data at 20 Hz on the x, y, and z axes. The wearable device transmits signals using Bluetooth Low Energy (BLE), and the Received Signal Strength Indicator (RSSI) values are logged at each gateway on packet arrival using the BLUEZ driver using a Broadcom BCM43438 combo Wi-Fi and BLE 4.1 System on Chip (SoC). It has a tabular structure containing all information of each epoch: timestamps, RSSI values, packet sequence numbers, accelerometer measurements, receiving gateway nodes, and labels indicating actual room locations and corresponding activities. The dataset targets the challenge of localization within smart homes with actions of sitting, lying, walking, and standing, among others, and locales such as kitchen, bedroom, living room, and stairs. The extensive dataset allows full assessment of joint localization and activity recognition. For more details about the dataset and the calibration process, readers are referred to [29].

We have relied on a single benchmark dataset to ensure a controlled and reliable evaluation of our proposed model. This decision is driven by several factors that limit the feasibility of using multiple datasets in the current study. There is currently a scarcity of publicly available datasets that provide fine-grained indoor location and human activity labels synchronized in time. Standard benchmarks such as PAMAP2 [31], Opportunity [32], and UCI HAR [33] focus solely on activity recognition. At the same time, datasets like UJIIndoorLoc [34] and IndoorLoc [35] are dedicated to localization without activity annotations, limiting their applicability for training or evaluating joint models. Additionally, existing datasets exhibit significant variability in sensor modalities (e.g., WiFi, BLE, IMU), deployment scenarios, room configurations, and sampling rates, which creates challenges for model transferability and alignment. Addressing this heterogeneity would require complex domain adaptation techniques, which fall outside the current study’s scope but represent an important avenue for future research. In this context, the EurValve SHiB dataset [29] stands out as it uniquely provides synchronized BLE-RSSI-based localization and wearable-derived activity recognition within a realistic smart home environment, enabling joint classification of activity-location pairs. This makes it particularly suitable for validating our proposed hybrid CNN-GRU-MRF architecture. This work aims to establish a robust methodological baseline capable of capturing spatial-temporal dependencies and inter-variable relationships, and using a single, well-characterized dataset ensures controlled benchmarking. It minimizes confounding variables that could obscure the methodological contributions.

## 4 Proposed hybrid CNN-GRU algorithm

The proposed hybrid CNN-GRU model combines convolutional neural networks (CNNs) for spatial feature extraction and gated recurrent units (GRUs) for temporal sequence modeling. This combination allows the complementary strengths of CNNs and GRUs to be used for efficient localization and activity classification. In this network, CNN layers extract hierarchical spatial features, while GRU layers capture temporal dependencies within the data. The final classification is achieved through a fully connected layer. The complete algorithm is given in Algorithm 1.

The CNN layers take the input data $x\in {\mathbb{R}}^{N\times T\times F}$, where *N* denotes the batch size, *T* represents the temporal length, and *F* denotes the feature dimension. The convolution operation is mathematically defined as

$$h_{\text{cnn}}=\text{ReLU}(\text{Conv2D}(x)+b),$$ (1)

where Conv2D(x) denotes the convolution operation applied to the input *x*, and *b* is the bias term. ReLU introduces non-linearity, making sure that the network has the ability to learn complex spatial patterns. Batch normalization followed by max-pooling is applied after each convolutional layer to stabilize training and reduce spatial dimension.

The temporal features extracted by the CNN are fed into the GRU layers. The GRU layers are bidirectional, capturing forward and backward dependencies in the temporal sequence. For each time step *t*, the GRU’s hidden state is updated using

$$h_{\text{gru}}^{\left(t\right)}=z^{\left(t\right)}⊙h_{\text{gru}}^{\left(t-1\right)}+(1-z^{\left(t\right)})⊙{\tilde{h}}^{\left(t\right)},$$ (2)

where ${\text{z}}^{\left(\text{t}\right)}$ is the update gate controlling the incorporation of new information, and ${\tilde{h}}^{\left(t\right)}$ is the candidate hidden state, computed as

$${\tilde{h}}^{\left(t\right)}=\text{tanh}(W_{h}x^{\left(t\right)}+U_{h}(r^{\left(t\right)}⊙h_{\text{gru}}^{\left(t-1\right)})+b_{h}),$$ (3)

Here, ${\text{r}}^{\left(\text{t}\right)}$ is the reset gate, *W*_*h*_ and *U*_*h*_ are learnable weights, and *b*_*h*_ is the bias. The GRU effectively models temporal relationships, enhancing the sequence representation. The final classification is done through a fully connected layer with a softmax activation function given by

$$\hat{y}=\text{Softmax}(W_{\text{fc}}h_{\text{gru}}+b_{\text{fc}}),$$ (4)

where $W_{\text{fc}}$ and $b_{\text{fc}}$ are the weight matrix and bias term of the fully connected layer. The softmax function converts the logits into class probabilities. To ensure stable and efficient training, the model’s weights are initialized by using method introduced by Kaiming He *et al*. [36], defined as

$$W\sim 𝒰\left(-\sqrt{\frac{6}{n_{\text{in}}}},\sqrt{\frac{6}{n_{\text{in}}}}\right),$$ (5)

where $n_{\text{in}}$ denotes the number of inputs to the layer. This initialization preserves the variance of activations across layers, preventing gradient vanishing or explosion.

During training, the model optimizes the cross-entropy loss function given by

$$L=-\frac{1}{N}\sum_{i=1}^{N}y_{i}\log({\hat{y}}_{i}).$$ (6)

where *y*_*i*_ is the ground truth class label and ${\hat{y}}_{i}$ is the predicted probability. The parameters are updated through a gradient-based optimizer with weight decay to avoid over-fitting.

**Algorithm 1. Hybrid CNN-GRU model with He initialization and training procedure.**

**Input:** Input data x, ground truth labels y, number of epochs E, learning rate η, weight decay *λ*, CNN layers *L*_*cnn*_, GRU layers *L*_*gru*_, number of classes C.

**Output:** Trained model model.

**Model Initialization:**

Define the HybridCNNGRU model with input channels, CNN hidden dimension, GRU hidden dimension, and number of classes.

**Initialize CNN layers:**

**for** *i* = 1 **to** *L*_*cnn*_ **do**

{For each CNN layer}

**end for**

**Initialize GRU layers:**

**for** *i* = 1 **to** *L*_*gru*_ **do**

{For each GRU layer} Initialize GRU layer with bidirectional configuration.

**end for**

Define fully connected layer for classification.

Apply He initialization for weights:


$$W\sim 𝒰\left(-\sqrt{\frac{6}{n_{\text{in}}}},\sqrt{\frac{6}{n_{\text{in}}}}\right)$$

where $n_{\text{in}}$ is the number of input units to the layer.

**Training Loop:**

**for** *epoch* = 1 **to** E **do**

**Training Phase:**

Set the model to training mode.

**for** each batch $\left(x_{i},y_{i}\right)$ in the training dataset **do**

Reshape *x*_*i*_ for CNN input.

Zero the gradients of the model parameters.

Perform a forward pass: *output* = *model*(*x*_*i*_).

Compute loss $L=\text{CrossEntropyLoss}(output,y_{i})$.

Perform backward pass: *L*.*backward*().

Update model parameters using the optimizer: optimizer.step().

Accumulate loss and compute accuracy.

**end for**

**Evaluation Phase:**

Set the model to evaluation mode.

**for** each batch $\left(x_{i},y_{i}\right)$ in the test dataset **do**

Reshape *x*_*i*_ for CNN input.

Perform a forward pass: *output* = *model*(*x*_*i*_).

Compute loss $L=\text{CrossEntropyLoss}(output,y_{i})$.

Update test loss and accuracy.

**end for**

**end for**

**Envulate the performance of model on test data**. Accuracy, Recall, F1 score, Precision

**Fig 2:**
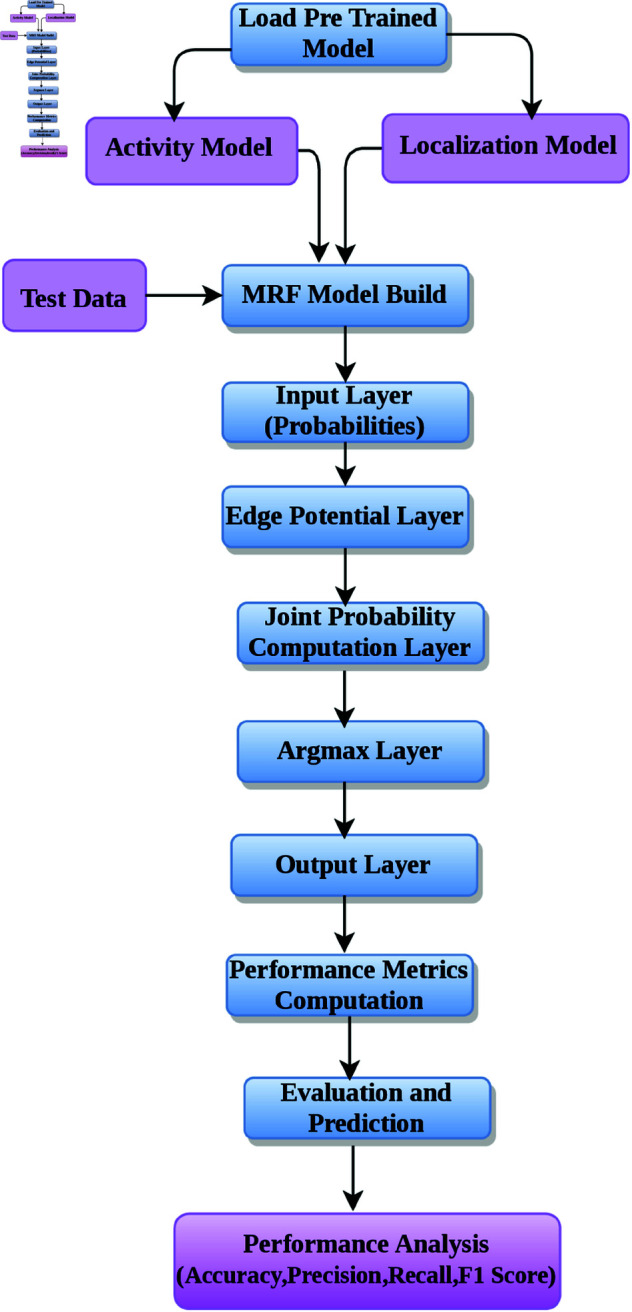
The proposed framework of MRF model.

This approach classifies the activities and locations first separately by using a hybrid CNN-GRU model. Then, it carrys out the classification of combined activity-location pairs by relying on the same CNN-GRU architecture but integrating MRF into the architecture to further model dependencies in activity-location pairs the proposed diagram is shown in Fig 2.

This combination enables the model to harness the spatial-temporal modeling provided by CNN-GRU and probabilistic inference enabled through MRF, which guarantees a robust classification of the activity-location pair. The MRF models the joint probability distribution of activities and locations by incorporating their compatibility using an edge potential matrix. An MRF is a graphical model that represents a set of random variables with an undirected graph, where nodes represent the variables (activity and location in this case), and edges encode their pairwise dependencies. In this context, the joint probability for an activity-location pair is expressed as

$$P(a,l)=\frac{1}{Z}P(a)P(l)\psi (a,l),$$ (7)

where *P*(*a*) and *P*(*l*) are the individual probabilities of activity *a* and location *l*, that are obtained from the softmax outputs of the CNN-GRU models for activity and location classification. These probabilities are then combined using the MRF framework to compute the joint probabilities *P*(*a*,*l*). The most probable activity-location pair is inferred using

$${\hat{y}}_{\text{joint}}=\arg\max_{a,l}P(a,l).$$ (8)

In (7) ψ(a,l) is the edge potential matrix representing the compatibility between *a* and *l*, and *Z* is the normalization constant ensuring the probabilities sum to one as

$$Z=\sum_{a,l}P(a)P(l)\psi (a,l).$$ (9)

The edge potential matrix ψ(a,l) is used to encode the compatibility between activities and locations. It is empirically estimated from the training data by analyzing the co-occurrence frequencies of activities and locations. More specifically, a 2D matrix *C* is built in which each cell *C*(*i*,*j*) denotes the number of occurrences of the activity *i* with location *j*. The matrix is then normalized to compute the probabilities as given by

$$\psi (i,j)=\frac{C(i,j)}{\sum_{k}C(i,k)}.$$ (10)

Here, $\sum_{k}C(i,k)$ denotes the total number of occurrences of activity *i* in all locations. This normalization ensures that the edge potentials are proper probabilities, summing to one for each activity.

To improve the description of the semantic and empirical correspondences between human activities and respective indoor spaces, we construct a compatibility matrix ψ(a,l). where each element represents the conditional likelihood of a specific activity occurring at a particular location. It is empirically learned from the training set frequency statistics about how often each activity-location pair is seen.

**Fig 3:**
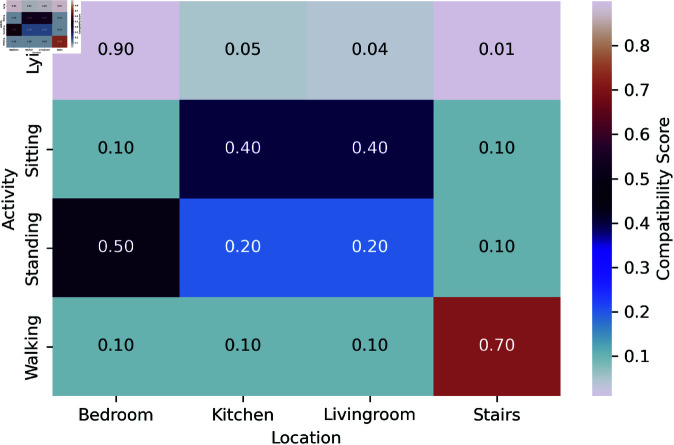
Activity location compatibility matrix.

The matrix is row-normalized for valid probability distributions over locations. The value for each activity adds up to one, for example. The model learns that lying is most affiliated with the bedroom (compatibility score: 0.90) while walking is most compatible with the stairs (0.70). Sit displays an even split between the kitchen and living room (0.40), as both are used for sitting activities.The compatibility matrix acts as the Markov Random Field (MRF) model’s edge potential function and is key in improving the joint activity-location inference. It regulates the model to make contextually plausible predictions and mitigate errors in ambiguity or noise by promoting high probability relations based on empirical patterns.We display this matrix as a heatmap Fig 3 to enhance interpretability, illustrating the learned relationships between activities and locations. This visualization assists in conveying the learned context-awareness of the model and aids in its improved comprehension of inferential mechanisms.

The MRF model in the proposed methodology operates by evaluating the trained activity and location classifiers in evaluation mode, the algorithm is given in 2.

**Algorithm 2. Activity and localization model inference.**

**Input:** Test data loaders test_loader_activity, test_loader_location, test_loader_combined, trained models activity_model, location_model, *mrf* model.

**Output:** Joint confusion matrix for activity-location pairs.

**Model Initialization:**

**Generate Probabilities:**

Do for each batch in test_loader_activity and test_loader_location:

**for** each batch $(inputs{,}_{)}\in test\_loader$ **do**

Apply softmax to model outputs for activity and location.

**end for**

**Generate Joint Probabilities using MRF:**

Do for each combined data batch:

**for** each batch (activity_data,localization_data,activity_label,localization_label)∈test_loader_combined **do**

Compute activity and location outputs using softmax.

Compute joint probabilities with MRF.

Infer most probable activity and location.

Store true and predicted labels.

**end for**

Encode joint labels and compute confusion matrix.

**Plot and Save Confusion Matrix:**

Plot the confusion matrix and classification report.

## 5 Result and discussion

The proposed approach and benchmark methodologies are evaluated using a confusion matrix with thorough explanations for each strategy. A complete categorization report for the three scenarios is provided, and sensitivity and specificity are discussed in the subsequent sections.

### 5.1 Scenario 1

**Fig 4:**
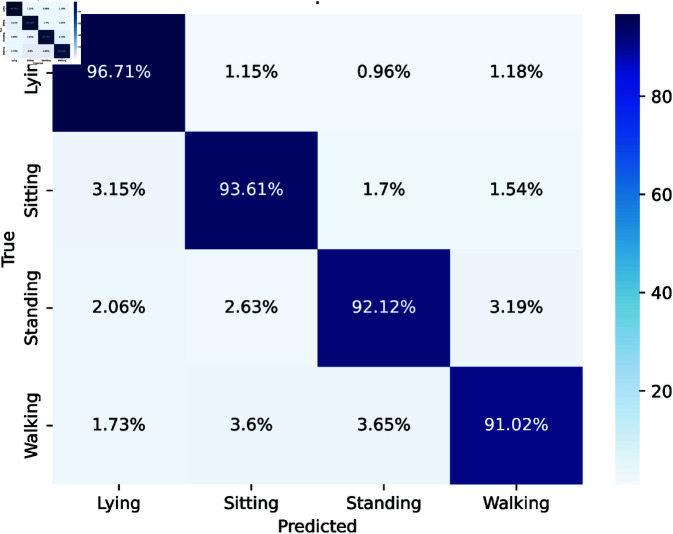
Confusion matrix of activity recognitions.

The confusion matrix for activity recognition shows that the model can identify activities with powerful temporal and spatial pattern learning and confusion matrix is given in Fig 4. Lying is most accurate 96.71% with few misclassifications. Sitting and standing perform well with 93.61% and 92.12% accuracy, respectively, though minor confusion occurs with adjacent activities. Walking is 91.02% accurate, often misclassified as standing or sitting.

The classification report of activity recognition is given in Table 1. Lying detects with low false negatives and 0.94 precision, 0.97 recall, and 0.95 F1 score. Sitting F1 score 0.94 suggests balanced accuracy owing to continuous precision and recall. However standing has 0.95 accuracy, 0.92 recall, and 0.93 F1 score, indicating more false negatives while walking exhibited equal accuracy and recall with an F1 score of 0.91, indicating reliable identification.

**Table 1:** Classification report for activity recognition.

Class	Precision	Recall	F1-Score
Lying	0.94	0.97	0.95
Sitting	0.94	0.94	0.94
Standing	0.95	0.92	0.93
Walking	0.91	0.91	0.91

### 5.2 Scenario 2

**Fig 5:**
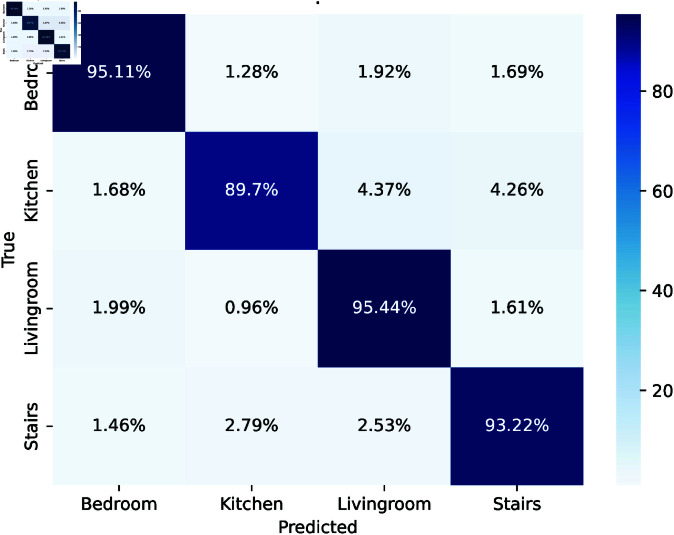
Confusion matrix of localization.

The confusion matrix evaluates interior localization in the bedroom, kitchen, living room, and stairwell is given in Fig 5. The elevated diagonal accuracy bedroom: 95.11%, living room: 95.44% indicates robust model predictions for certain regions. The few off diagonal errors like kitchen misclassified as bedroom: 1.68% indicate commendable accuracy with negligible overlaps. The matrix indicates that the proposed strategy is effective and recommends methods to enhance inter class uniqueness for improved localization accuracy.These findings are crucial for indoor intelligent system development.

The indoor localization classification report shows strong performance across four situations as given in Table 2. A precision of 0.96, recall of 0.95, and F1 score of 0.95 signify high accuracy with little false positives and negatives for bedroom. Kitchen achieved an accuracy of 0.92, a recall of 0.90, and an F1 score of 0.91, demonstrating dependable detection with potential for memory improvement. Livingroom achieved an accuracy of 0.93, a recall of 0.95, and an F1 score of 0.94, indicating proficient identification with slightly enhanced recall. Stairs exhibited steady performance with an accuracy of 0.94, a recall of 0.93, and an F1 score of 0.93.

**Table 2:** Classification report for indoor locations.

Class	Precision	Recall	F1-Score
Bedroom	0.96	0.95	0.95
Kitchen	0.92	0.90	0.91
Livingroom	0.93	0.95	0.94
Stairs	0.94	0.93	0.93

### 5.3 Scenario 3

**Fig 6:**
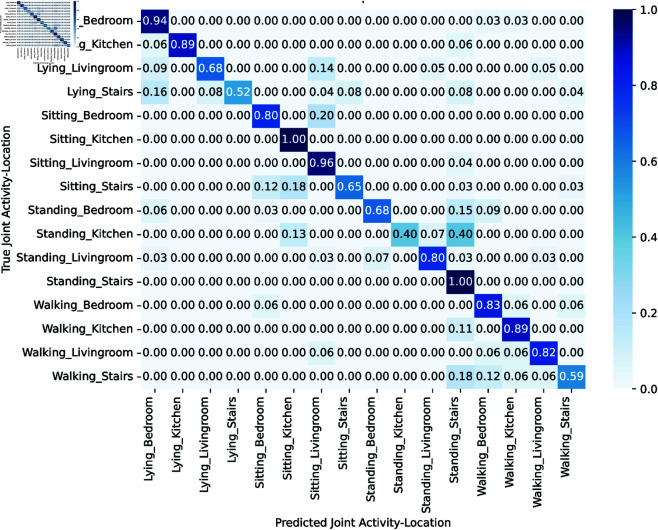
Confusion matrix of joint activity localization.

Activity recognition and indoor localization results are presented in this scenario. Fig 6 gives the confusion matrix of joint activity and localization. A Hybrid CNN-GRU is used to evaluate activities and locations separately, thereafter integrating the data using a MRF to ascertain joint probability. Insights from the joint activity-location confusion matrix about model performance and safety scenarios are essential. Recognizing hazardous events such as lying in kitchen 0.89 accuracy is essential, since they may signify a fall and need immediate emergency intervention. The accuracy of standing on stairs 1.0 demonstrates steadiness, whereas sitting on stairs 0.65 may suggest fatigue or pain. Walking in bedroom 0.83 is often acceptable however, errors in lying or sitting may suggest an anomaly. The lower accuracy for “walking on stairs” (0.59) indicates difficulties perceiving dynamic motion, increasing the danger of unexpected falls. Nonetheless, sitting in living Room 0.96 is readily identifiable. The consistency of activity and location predictions using MRF with Hybrid CNN-GRU enhances detection in high-risk situations and ensures indoor user safety.

The study on combined activity and location recognition classification demonstrates model intricacies as given in Table 3. Lying_Bedroom had a high F1-score of 0.83 and recall, while Lying_Kitchen attained 0.94 in precision. However Lying_Stairs exhibited a decreased F1 score 0.68 while maintaining excellent precision 1.00. Sitting_Kitchen and Standing_Livingroom had commendable F1 scores of 0.81 and 0.86, respectively, indicating precise detection. The data indicate that the model can classify activities and locations concurrently with variability across specific scenarios and conditions.

**Table 3:** Joint activity-location classification report.

Class	Precision	Recall	F1-Score
Lying_Bedroom	0.75	0.94	0.83
Lying_Kitchen	1.00	0.89	0.94
Lying_Livingroom	0.88	0.68	0.77
Lying_Stairs	1.00	0.52	0.68
Sitting_Bedroom	0.73	0.80	0.76
Sitting_Kitchen	0.68	1.00	0.81
Sitting_Livingroom	0.69	0.96	0.80
Sitting_Stairs	0.92	0.65	0.76
Standing_Bedroom	0.92	0.68	0.78
Standing_Kitchen	1.00	0.40	0.57
Standing_Livingroom	0.92	0.80	0.86
Standing_Stairs	0.49	1.00	0.66
Walking_Bedroom	0.68	0.83	0.75
Walking_Kitchen	0.67	0.89	0.76
Walking_Livingroom	0.82	0.82	0.82
Walking_Stairs	0.77	0.59	0.67

The high precision for Lying_Kitchen and Standing_Livingroom indicates robust classification accuracy. Reduced recall in Lying_Stairs and Standing_Kitchen indicates difficulties in remembering all pertinent events, perhaps signifying emergency risks.

The F1 scores effectively balance accuracy and recall, demonstrating strong performance in Lying_Bedroom and Sitting_Livingroom. In critical situations such as Walking_Stairs and Standing_Stairs, low scores indicate potential for enhancement in high-risk, transitional activities. Our results indicate that more refinement is necessary for successful activity-location identification, especially in safety-related scenarios.

### 5.4 Scenario 4

To demonstrate the effectiveness of the Markov Random Field (MRF) inclusion in the proposed framework, we perform the activity and location classification without MRF first, as shown in Table 4. The results indicate that although the multi-task CNN-GRU model achieves a reasonable level of effectiveness in the task of isolative activity classification (e.g., F1-scores of 0.91 for Sitting and 0.90 for Lying), its location classification is much poorer—especially in settings with semantic ambiguity or poor signal strength, such as Kitchen (F1-score: 0.097) and Bedroom (F1-score: 0.274), as compared to the results with MRF (shown in Table 3). This result indicates that joint learning of activities and locations without explicitly representing their relationships might cause misaligned or inconsistent predictions. In contrast, the proposed CNN-GRU+MRF model uses a learned edge potential matrix ψ(a,l) to capture contextual compatibility, which results in much better joint classification performance (e.g., Lying_Bedroom: F1 = 0.83, Sitting_Kitchen: F1 = 0.81, Standing_Livingroom: F1 = 0.86).

**Table 4:** Classification report for activity and location classification using the multi-task learning model.

Activity Classification					Location Classification				
Activity Class	Precision	Recall	F1-Score	Accuracy	Location Class	Precision	Recall	F1-Score	Accuracy
Lying	0.9003	0.9093	0.9048	0.9093	Bedroom	0.2800	0.2684	0.2741	0.2684
Sitting	0.9112	0.9268	0.9189	0.9268	Kitchen	0.1692	0.0680	0.0970	0.0680
Standing	0.8614	0.8660	0.8637	0.8660	Livingroom	0.2886	0.3302	0.3080	0.3302
Walking	0.8620	0.8183	0.8396	0.8183	Stairs	0.2656	0.3415	0.2989	0.3415

Combining Convolutional Neural Networks and Gated Recurrent Units (CNN-GRU) with MRF remedies a fundamental shortcoming in joint activity and localization models: the absence of contextual information. Although CNN-GRU successfully extracts spatial-temporal dynamics, the MRF module integrates probabilistic inference over activity-location correspondences through ψ(a,l). This produces more semantically consistent and noise-resistant predictions, particularly in noisy real-world scenarios or overlapping pattern behavior. Generally, CNN-GRU+MRF proposes a better contextual and interpretable joint inference technique with an efficient application in innovative healthcare, ambient assisted living, and intelligent indoor environments.

## 6 Limitations

The following text highlights some of the limitations of the proposed work. Scalability to Additional Classes: Although our system demonstrates acceptable results with the present collection of four activities and four indoor locations, scaling to additional classes may bring more inter-class overlap and might call for retraining or fine-tuning the architecture. We recognize that the more activity-location combinations there are, the higher the model complexity and the data demands will be.

Sensitivity to Sensor Dropout: The system depends on continuous data from the gateway and wearable sensors. Sensor dropout or latency in communication can impact model performance, especially for real-time deployment. As a countermeasure, we plan to incorporate learning mechanisms of dropout robustness and data imputation techniques in future research.

Ambiguous Activity-Location Pairs: Some activity-location pairs—e.g., sitting in the kitchen or lying on stairs—are semantically ambiguous or naturally rare. Such instances can cause classification ambiguity. Our MRF model has mitigated this somewhat by capturing co-occurrences. Still, we acknowledge that context-dependent priors or multimodal fusion (e.g., vision or ambient sensors) are needed to disambiguate such instances further.

Generalization Across Environments: The new model has been tested with data from a particular smart home setup. The performance is promising in this restricted setting, but direct extrapolation to other layouts, building materials, and sensor positions in actual homes or hospitals may lead to less accurate results. Transfer learning and domain adaptation methods must be investigated in future research to tackle cross-environment robustness.

## 7 Conclusion

This paper proposed a hybrid CNN-GRU and MRF-based framework for the joint classification of indoor localization and human activities. The system leverages the spatial-temporal strengths of CNN-GRU for individual classifications and enhances the consistency of prediction by using the MRF framework. Experimental evaluations have shown substantial improvement in classification accuracy for both tasks with achieved precision and recall metrics befitting practical applications in healthcare and smart home systems. The proposed framework is especially applicable in real-world applications of smart homes, healthcare systems, and IoT-based environments where precise activity-location prediction is essential. Particularly, the combined activity-location classification pointed out the potential of integrating probabilistic dependencies for enhancing overall model reliability. These results verify the effectiveness of our approach and its applicability in environments requiring accurate and dynamic user context understanding. Future endeavors include augmenting datasets for variety, including modalities such as audio and ambient sensors, enhancing compute efficiency for edge devices, and investigating semi-supervised learning to diminish dependence on labeled data. The additions seek to augment the flexibility, scalability, and efficiency of the CNN-GRU-MRF framework for wider real-world applications.
